# Quantitative DNA metabarcoding reveals species composition of a macrocyclic lactone and pyrantel resistant cyathostomin population in the UK

**DOI:** 10.1016/j.ijpddr.2024.100576

**Published:** 2024-12-22

**Authors:** K.E. Bull, J. Hodgkinson, K. Allen, J. Poissant, L.E. Peachey

**Affiliations:** a Bristol Veterinary School, University of Bristol, Bristol, BS40 5DU, UK; b Department of Infection and Microbiome, Institute of Infection, Veterinary and Ecological Sciences, Leahurst Campus, University of Liverpool, Neston, CH64 7TE, UK; c Faculty of Veterinary Medicine, University of Calgary, 3280 Hospital Drive NW, Calgary, AB, T2N 4Z6, Canada

**Keywords:** Nemabiome, ITS-2, Cyathostomins, Thoroughbred, Resistance, Anthelmintics, Faecal egg count

## Abstract

Cyathostomins are the most abundant equid endoparasites globally. There are approximately fifty cyathostomin species and, whilst they occupy distinct niches within the large intestine, they are generally considered to share similar characteristics in terms of pathogenicity and response to drug treatment. There are three classes of anthelmintic licensed in the UK to treat cyathostomins (benzimidazoles, tetrahydropyrimidines and macrocyclic lactones) and cases of resistance have been documented for all classes. Previously, faecal egg count reduction tests (FECRT) on four UK Thoroughbred studs revealed multidrug resistant cyathostomins on one stud (A), with evidence of resistance to the macrocyclic lactones (MLs) ivermectin (IVM) and moxidectin (MOX), and to pyrantel (PYR). The remaining three studs (B-D) lacked resistance to IVM and MOX but had a shortened egg reappearance period post treatment.

To determine whether specific species could be associated with the observed resistance and shortened egg reappearance period, strongyle eggs collected from between six and 15 individual horses per stud were copro-cultured to third larval stage (L3), before and after anthelmintic treatment, over a three-year timeframe (2021–2023). Quantitative DNA metabarcoding of the ITS-2 region was carried out on all samples.

On stud A, single but differing species were found to be responsible for ML and pyrantel resistance in yearlings, *Cyathostomum catinatum* and *Cylicocyclus nassatus,* respectively. On studs B-D, with shortened egg reappearance periods, species composition remained largely unchanged post treatment.

This study is the first to quantitatively profile cyathostomin species composition pre- and post-treatment in a multidrug resistant population in the UK, revealing that resistance in cyathostomins was species specific. This raises the question of whether these species may be responsible for ML and PYR resistance more widely and indicates that anthelmintic resistance in cyathostomins may not be a multi-species phenomenon.

## Highlights

• ITS-2 metabarcoding revealed cyathostomin species proportions pre- and post-drug treatment on four studs.
• *Cya. catinatum* comprised between 97.5 and 99.5% of eggs shed in the ML-resistant population two weeks post ML treatment.
• *Cyl. nassatus* was more abundant after PYR treatment in the PYR-resistant population.
• Non-ML resistant populations had no significant changes in species abundance post treatment.
• A shortened ERP is associated with the presence of multiple cyathostomin species post treatment.

## Graphical abstract

**Figure:**
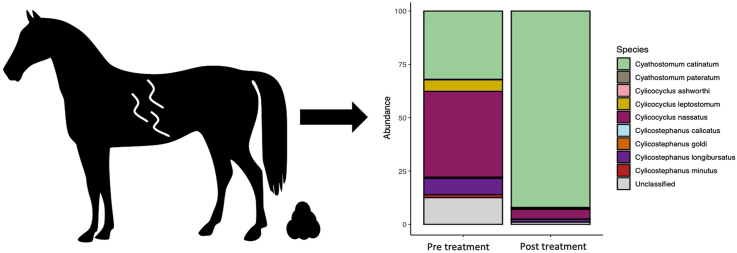

## 1 Introduction

Cyathostomins are the most abundant endoparasite species to infect equids globally (Corning, 2009). In horses with high infection levels, these parasites have the potential to cause larval cyathostominosis. This is characterised by mass emergence of cyathostomin larvae from the gut mucosa into the intestinal lumen, with clinical signs including protein losing enteropathy, diarrhea, weight loss and colic and a reported mortality rate of up to 50% (Love et al., 1999; Lyons et al., 2000; Lawson et al., 2023). Immune compromised individuals (Reid et al., 1995) and youngstock (Lawson et al., 2023) are most at risk of significant pathology.

Benzimidazoles (BZs, e.g., fenbendazole, FBZ), tetrahydropyrimidines (e.g., pyrantel, PYR) and macrocyclic lactones (MLs, e.g., ivermectin (IVM) and moxidectin (MOX)) are the three classes of anthelmintics that are licensed in the UK to treat against cyathostomins, with MOX (at 0.4 mg/kg single dose) and FBZ (at 7.5 mg/kg for five days) the only drugs labelled for larvicidal efficacy. Reports of anthelmintic resistance to FBZ are widespread; 58 studies across 31 countries looked at BZ efficacy on equine strongylids since the year 2000 and all 58 reported anthelmintic resistance (Nielsen, 2022) including feral horse populations (Kuzmina et al., 2020). Reports of resistance to PYR are rapidly increasing; efficacy of PYR was tested in 37 studies since the year 2000, with 34 reporting resistance (Nielsen, 2022). Reports of suspected resistance to the MLs are still in low numbers and usually attributed to one animal in the group or borderline anthelmintic resistance (Canever et al., 2013; Relf et al., 2014; Traversa et al., 2009). However, recently there have been confirmed cases for both IVM and MOX in the USA (Nielsen et al., 2020), Australia (Abbas et al., 2021), the UK (Bull et al., 2023) and France (Merlin et al., 2024). Given the frequent movement of horses it is unlikely that these are isolated cases and the spread of ML resistant cyathostomins is a significant threat to equine populations.

Whilst there have been indications that different species occupy different niches in the gastrointestinal tract (Stancampiano et al., 2010; Morariu et al., 2016; Byrne et al., 2024) and may have differences in pathogenicity, cyathostomin species are generally considered to share similar characteristics. There is a lack of information on whether anthelmintics are equally efficacious against all cyathostomin species, due to the historic difficulty in quantitively differentiating the 51 cyathostomin species (Lichtenfels et al., 2008). The original method to identify cyathostomin species relied on morphological analysis of adults, a time-consuming process that relied on collection of adults post-mortem or from faeces post-treatment (Lichtenfels et al., 2008). Based on the data generated by morphological identification of adult worms, the most common species are considered to be *Cyathostomum catinatum*, *Cylicocyclus nassatus* and *Cylicostephanus longibursatus* (Bellaw and Nielsen, 2020)*.* The development of methods such as the species-specific PCR-enzyme-linked immunosorbent assay (PCR-ELISA), which detects 6 common species (Hodgkinson et al., 2003, 2005) and reverse-line blot (RLB) assays, which detect 21, 13 and 12 of the cyathostomin species that infect horses (Traversa et al., 2007; Ionita et al., 2010; Cwiklinski et al., 2012) paved the way for molecular-based species identification of easily accessible parasite stages.

Over the last decade a novel quantitative metabarcoding molecular method using the nuclear second internal transcribed spacer (ITS-2) rDNA has been developed for gastrointestinal helminths, generally known as nemabiome sequencing (Avramenko et al., 2015). Recently, this technique has been optimised to quantify common strongyles of horses (Poissant et al., 2021), facilitating studies exploring cyathostomin species composition. Since the optimisation of this tool, it has been used in a number of different studies exploring species composition in different populations Including a longitudinal study in Scottish ponies (Sargison et al., 2022) and species richness data for countries or various geographical locations (Mitchell et al., 2019; Nielsen et al., 2022; Sargison et al., 2022; Abbas et al., 2023; Halvarsson et al., 2024; Hamad et al., 2024). The approach is also beginning to be used to study anthelmintic resistance (Nielsen et al., 2022; Hedberg Alm et al., 2023; Abbas et al., 2024; Halvarsson et al., 2024); for example, a case of reduced egg reappearance period (ERP) in IVM and MOX in the USA found Cya. catinatum and Cyl. nassatus were the dominant species post MOX treatment and *Cyathostomum* insigne, Cylicocyclus radiatus, Cya. catinatum, Cylicocyclus elongatus and Cyl. nassatus were the species present at two weeks following IVM treatment (Nielsen et al., 2022). On the other hand, in 19 Swedish farms, of which 9 had pyrantel resistance and 7 were susceptible, treatment was found not to significantly affect species composition (Hedberg Alm et al., 2023). To our knowledge this tool has only been applied to one case of confirmed anthelmintic resistance to IVM in Australia (Abbas et al., 2024). This study profiled species abundance following administration of a number of anthelmintic drugs; the predominant species post treatment varied with the drug, with *Cyl. nassatus* and *Cyl. longibursatus* dominant post IVM, and *Coronocyclus Coronatus* and *Cylicostephanus Calicatus* dominant post MOX. It is important to establish whether AR is a multi-species phenomenon, or whether it is attributed to particular species, as this will determine the utility of the nemabiome tool to inform strategic drug use in cases of anthelmintic resistance. Hence our study aimed to apply nemabiome sequencing to establish which cyathostomin species are responsible for the first case of ML resistance in the UK (Bull et al., 2023).

## 2 Material and methods

### 2.1 Parasite populations and resistance status

Faecal samples were collected from horses on four Thoroughbred (TB) studs in England (A-D), with between six and 15 individuals from each stud sampled over a three year period (2021, 2022 and 2023; Table 1). Detailed results of fecal egg count reduction tests (FECRTs) were published previously in Bull et al. (2023). These results showed that stud A had cyathostomin populations in yearling horses that were resistant to IVM, MOX and PYR, and studs B,C,D had shortened ERP ranging from 4 to 7 weeks for IVM and 6 weeks for MOX (Bull et al., 2023). For reference the raw faecal egg counts (FECs) and pooled FEC values are shown in Supplemental Table 1 as these were used to calculate an approximate FEC for each species (see section 2.6).

**Table 1:** Details of UK studs included in this study, including the age group, number of horses sampled, date of treatment, drug used, egg reappearance period (ERP) and number of species identified using DNA metabarcoding before and after treatment. Number of species of cyathostomins post treatment is specific to the first post treatment sample sequenced that reached at value of greater than 10% of pre treatment FEC.

Stud	Group (yearlings (Y)/mares(M))	Number of horses in group	Treatment date	Drug	Resistant (Yes or No)	Egg reappearance period (ERP)^a^	Number of species of cyathostomins pre treatment	Number of species of cyathostomins post treatment
A	Y	11	Mar-21	IVM + PRZ	Y	N/A	9	2
A	Y	11	June-21	MOX	Y	N/A	7	6
A	Y	10	Aug-21	PYR	Y	N/A	7	8
A	Y	15	Jan-22	IVM + PRZ	Y	N/A	9	7
A	M	11	Apr-21	IVM + PRZ	N	7	9	3
A	M	10	July-21	MOX + PRZ	N	10	8	6
B	Y	14	May-21	MOX + PRZ	N	6	9	9
B	Y	6	June-22	IVM	N	7	9	7
C	Y	10	June-21	IVM	N	4	8	8
C	Y	15	Mar-22	MOX + PRZ	N	6	12	9
D	Y	13	May-22	MOX	N	6	8	7
D	Y	9	June-23	IVM	N	4	9	10

a N/A indicates that a positive egg count was recorded at 1 or 2 weeks after treatment and hence an ERP did not apply.

### 2.2 Larval culture and lysis

Fresh faeces from an individual horse at each sampling timepoint was mixed with equal part vermiculite and incubated at 25 °C for 14 days for L3 coproculture. Using the method from Poissant et al. (2021), with minor modifications to increase the amount of faeces cultured, L3 were collected from individual samples using the glass-over-petri-dish method (Dunn, 1978). Aliquots of equal numbers of L3 were taken from each individual horse sample and combined in one tube to total 2000–4000 L3 i.e., 200–400 L3 from 10 individual faecal samples, since they grazed the same pasture, they were all exposed to the same parasite challenge and assumed to harbor the same cyathostomin population. L3 were then left to settle at 4^∘^C for 1 h, transferred to a 15 ml falcon tube and centrifuged at 4500 g for 5 min at 0^∘^C. Supernatant was removed without disturbing the 1.5 ml sediment and centrifuged at 13000 g for 3 min. The supernatant was removed with a pipette down to 300 μl, following which 700 μl of 100% molecular grade ethanol was added. The samples were stored in 4∘C until processing.

The following methods were replicated from the protocol of previous research on the nemabiome of both sheep and horses (Avramenko et al., 2015; Poissant et al., 2021). In brief, surplus ethanol was removed and lysis buffer was added to the 4000 ethanol-fixed L3s and incubated at room temp for 5 min (recipe for lysis buffer in Avramenko et al., 2015). L3 were then centrifuged, and supernatant was removed followed by suspension of the pellet in lysis buffer. This cleaning step was repeated three more times and the supernatant was removed to leave ∼100 μl volume. The pellet was re-suspended and another 50 μl of lysis buffer was added. L3s were then placed on thermomixer for 15 min at 95∘C, with shaking at 1000 RPM. Following this they were placed at −80∘C for ∼12 h. The sample was then de-frosted on ice and 6 μl Proteinase K (20 mg/ml) was added to each sample to achieve a final conc of 0.8 mg/ml. The sample was then placed on thermomixer at 55∘C for minimum of 12 h, with shaking at 800 RPM followed by 20 min at 95∘C to denature the Proteinase K. It was then placed directly on ice and the lysate was diluted with molecular grade water to 1:10 was used as the template for PCR. The lysate and lysate dilution were stored at −80∘C.

### 2.3 ITS2 amplification and Illumina MiSeq sequencing

Methods to amplify the ITS2 gene region were taken from previously published work by Avramenko et al. (2015). NC1 and NC2 primers were used (Gasser et al., 2008) with up to three random bases (N) to increase amplicon diversity and an adapter sequence was added to allow incorporation of barcode in the second PCR. DNA template consisted of 21ul of mastermix and 4ul of worm lysate dilution which was added to each well for the PCR. Mastermix consisted of 5ul KAPA HiFi HotStart Fidelity Buffer (5X) (KAPA Biosystems, USA), 0.75ul NC1 and NC2 primers (10 μM), 0.75ul dNTPs (10 mM), 0.5ul KAPA HiFi HotStart Polymerase and 13.25ul water per sample. Blank controls were included on each plate containing water instead of DNA lysate. Three plates of samples were sequenced, 40 samples on plate one were pooled together, 19 samples on plate two and 13 samples on plate three. For the amplicon PCR the following cycling conditions were used: 95 °C for 2 min followed by 25 cycles of 98 °C for 20 s, 62 °C for 15 s and 72 °C for 15 s and then a single step of 72 °C for 2 min. PCR product was run through an electrophoresis gel to ensure DNA was amplified prior to sequencing. PCR product was purified using AMPure XP magnetic beads (Beckman Coulter, Inc., Brea, California). A second PCR was run using 20 Illumina barcodes using the following conditions: 98 °C for 45 s, followed by seven cycles of 98 °C for 20 s, 63 °C for 20 s, 72 °C for 2 min (Avramenko et al., 2015). PCR product was again purified using AMPure XP magnetic beads (Beckman Coulter, Inc.) and 50 ng of DNA from each sample was pooled to prepare a library which was quantified using a qPCR. The pooled library at 12.5 nM concentration was sequenced on an Illumina MiSeq with a v2 500-cycle reagent kit following Illumina's standard MiSeq operating protocol to produce FASTQ files.

### 2.4 Processing ITS2 amplicons

The following methods to process amplicon reads and assign taxonomy were replicated using updated code from previously published work (Poissant et al., 2021) available at https://data.mendeley.com/datasets/vhyysw8xt2/2. Demultiplexed reads were processed using DADA2 (version 2.22.0) to assign taxonomy (Callahan et al., 2016). Cutadapt (version 4.2) was used to remove primer sequences (Martin, 2011). The filterAndTrim function (maxN = 0) was used to remove all sequences containing ambiguous bases (Ns) and to discard reads that had ≥2 maximum expected errors in the forward direction or ≥ 5 in the reverse direction, sequences with a length less than 200 bp and bases with a quality score of two or less were also removed. Remaining reads were then used to construct an error model using the learnErrors command with a maximum of 15 rounds of convergence for building the error model (MAX_CONSIST). Error correction was conducted with the dada command using pool = TRUE. Corrected forward and reverse reads were then merged using mergePairs while allowing up to 2.5% base mismatches in the overlapping region, this value has been used in previous equine nemabiome work (Nielsen et al., 2022). All ASVs above the cutoff were removed.

### 2.5 Taxonomic assignment

Taxonomy assignment used the ITS2 database curated by Poissant et al. (2021) (version 1.3) and was assigned using the DADA2 assignTaxonomy command with the following parameters: minBoot = 0, tryRC = TRUE, outputBootstraps = TRUE. Species were assigned with at least 80% bootstrap support. The detection limit was set to 1/4000 to remove species that were only detected once in 4000 to reduce inclusion of false positives from index hopping (Wright and Vetsigian, 2016). This value was determined as this was the number of larvae pooled in each sequenced sample.

### 2.6 Statistical and bioinformatic analysis

R (Team, 2023) was used to analyse the raw reads, plot species abundance in stacked barplots and line graphs from the CSV files using the following packages: ‘ggplot2’ (Wickham, 2016) and ‘tidyr’ (Wickham et al., 2024). The FEC and species abundance data was used to plot the line graphs by using the relative abundance of each species and the pooled FEC to give an approximate FEC of each species pre and post treatment. Species abundance data from nemabiome sequencing was used to convert total FEC data into interpolated species-specific egg counts which was used to do species specific FECRT's on stud A using the WAAVP guidelines (Kaplan et al., 2023) and eggCounts package (Torgerson et al., 2014; Wang et al., 2018) (Table 2). It was only possible to do this on stud A as it was the only stud with a positive FEC (and hence metabarcoding data from larvae) at two-weeks post treatment.

**Table 2:** Faecal egg count reduction results for individual cyathostomin species using eggCounts package at week 0 and week 2 for Stud A.

Sample	Mean FEC-0	Mean FEC-2	% reduction	Mean *Cya.catinatum* FEC-0	Mean *Cya.catinatum* FEC-2	% reduction	Mean *Cyl.nassatus* FEC-0	Mean *Cyl.nassatus* FEC-2	% reduction
**A-IVM-Y**	767.5	503.5	40% (19.7–66.8%)	452.83	498.47	37.6% (20.3–67.5%)	184.2	0.50	99.8% (99.5–100%)
**A-MOX-Y**	324.55	98.18	72.6% (50.8–85.2%)	204.46	95.24	59.1% (33–77.9%)	6.49	3.93	48.8% (20.1–72.3%)
**A-PYR-Y**	829	103.5	80.% (62.9–90%)	165.8	0.93	99.4 (98.6–99.8%)	431.08	87.98	66.7% (40.1–83.2%)
**A-IVM-Y-Jan22**	1156.82	305	76.3% (64.5–84.8%)	370.18	280.6	34.9% (17.1–59.4%)	462.73	15.25	96.8% (94.7–98.1%)

Of note, the post treatment species results for the mares on stud A at weeks one, two and three are only from two individuals at timepoint one, and from three individuals at timepoint two and three, all other individuals in the group had FECs too low to culture.

### 2.7 Data accessibility

Raw sequences are available at Mendeley Data (https://doi.org/10.17632/9k2p24f9p6.1).

## 3 Results

The number of paired reads generated by the sequenced samples ranged from 9957 to 79,595 and between 9693 and 76,970 pairs/amplicons remained after quality filtering, denoising, merging, and chimera removal (Supplementary file1). Across the 3 plates there were 476 amplicon sequence variations (ASVs) assigned by DADA2, 375 were assigned to a genus and 362 to a species with at least 80% bootstrap support.

There were 12 cyathostomin species seen in the yearlings on the four studs sampled, nine on stud A, eight on stud B, 12 on stud C and eight on stud D with the most prevalent species across all samples being *Cya. catinatum, Cyl. nassatus* and *Cyl. longibursatus* (Table 1). Across the four studs the number of ASVs for a species with at least 80% bootstrap support was between 1 and 105. *Cya. catinatum* had 75 ASVs*, Cyl. nassatus* had 43 ASVs and *Cyl. longibursatus* had 105 across the four studs.

**Fig. 1:**
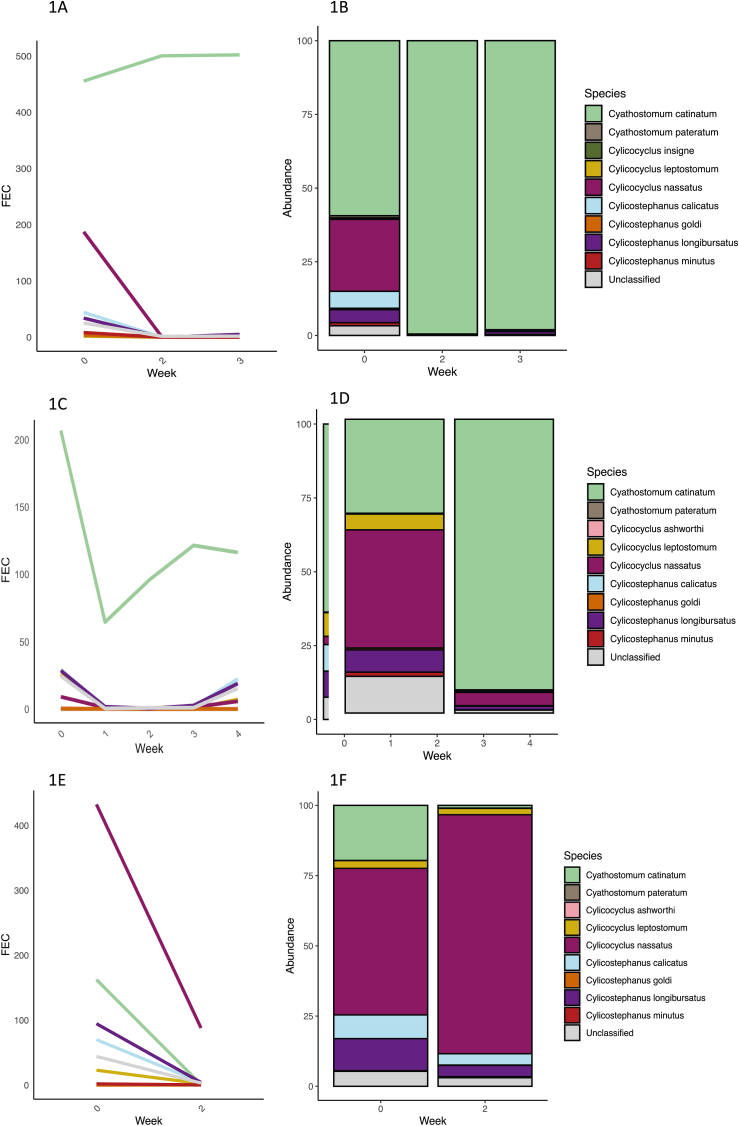
Cyathostomin species in yearlings on stud A in 2021 before and up to 4 weeks following ivermectin and moxidectin treatments inferred from DNA metabarcoding of pool of cultured L3 larvae. The line graph shows the estimated change in FEC for each species and the stacked bar plot shows species abundance.1A and 1B- stud A yearlings treated with ivermectin. 1C and 1D-stud A yearlings treated with moxidectin. 1E and 1F- stud A yearlings treated with pyrantel.

**Fig. 2:**
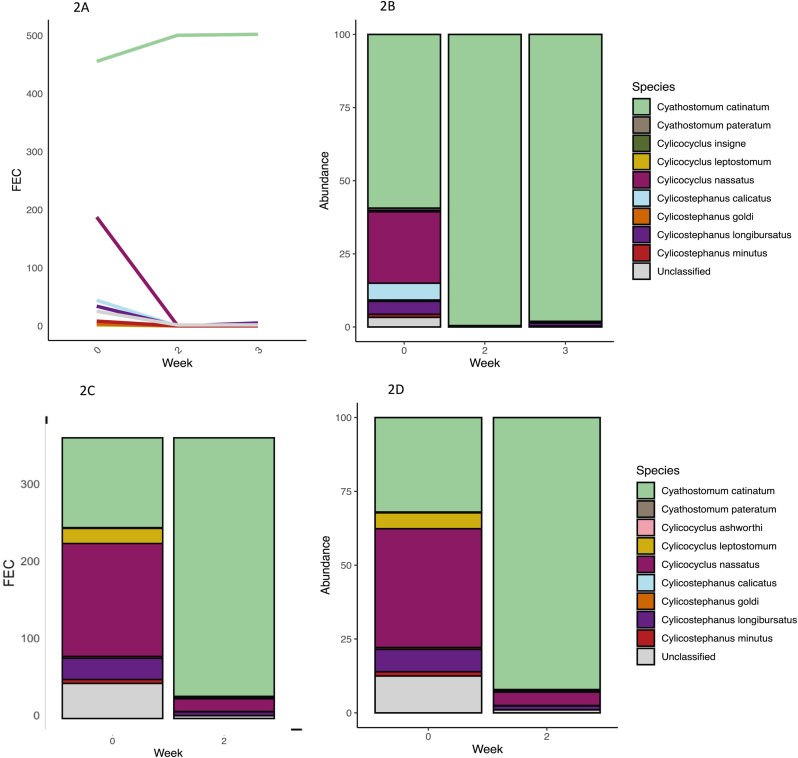
There is an apparent transfer of AR resistant cyathostomins between cohorts. The line graph shows the change in FEC for each species and the stacked bar plot shows species abundance. 2A and 2B- cohort one, stud A yearlings treated with ivermectin in March 2021.2C and 2D-cohort two, stud A yearlings treated with ivermectin in January 2022.

**Fig. 3:**
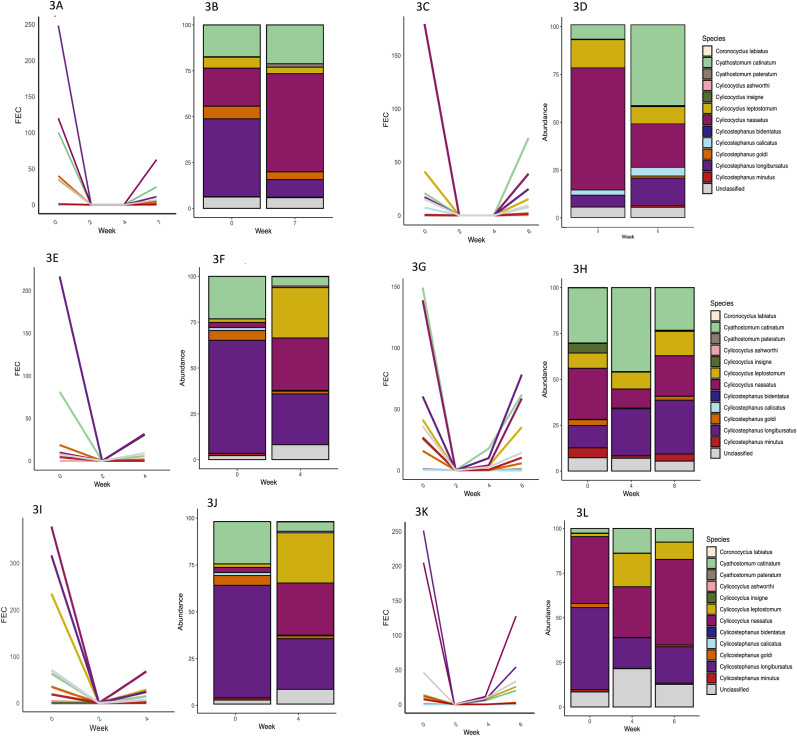
Cyathostomin species associated with the early egg reappearance on stud B, C and D in yearlings. The line graph shows the estimated change in FEC for each species and the stacked bar plot shows species abundance. 3A and 3B- stud B yearlings treated with ivermectin. 3C and 3D-stud B yearlings treated with moxidectin. 3E and 3F- stud C yearlings treated with IVM. 3G and 3H- stud C yearlings treated with moxidectin. 3I and 3J-stud D yearlings treated with IVM. 3K and 3L-stud D yearlings treated with moxidectin.

The species data for stud A, known to have cyathostomin populations resistant to IVM, MOX and PYR, showed that *Cya. catinatum* was the dominant species in resistant populations post IVM and MOX treatment (Fig. 1). This result was seen in both the 2021 and 2022 yearling cohorts (Fig. 2). In the post ML treatment samples *Cya. catinatum* constituted between 92.1% and 99.5% of the remaining cyathostomins species, compared to 31.9%–63.6% pre-treatment. When converting relative abundance to approximate egg counts, *Cya. catinatum* showed either no or very little reduction in FEC post treatment (Fig. 1, Fig. 2). On stud A, post PYR treatment it was *Cyl. nassatus* that appeared to be the predominant species (Fig. 2). On studs B, C and D there was no IVM or MOX resistance, but all three studs had a shortened egg reappearance period (ERP) compared to the original label claims for MLs. These studs did not have one predominant species post treatment, with a broadly similar mix of species evident pre and post treatment (Fig. 3). Species level FECR calculations were done on stud A data to look at individual species abundance pre and post treatment (Table 2). The results of this showed *Cya. catinatum* to be resistant to IVM and MOX treatments and *Cyl. nassatus* to be resistant to PYR treatment (Fig. 1, Fig. 2).

**Fig. 4:**
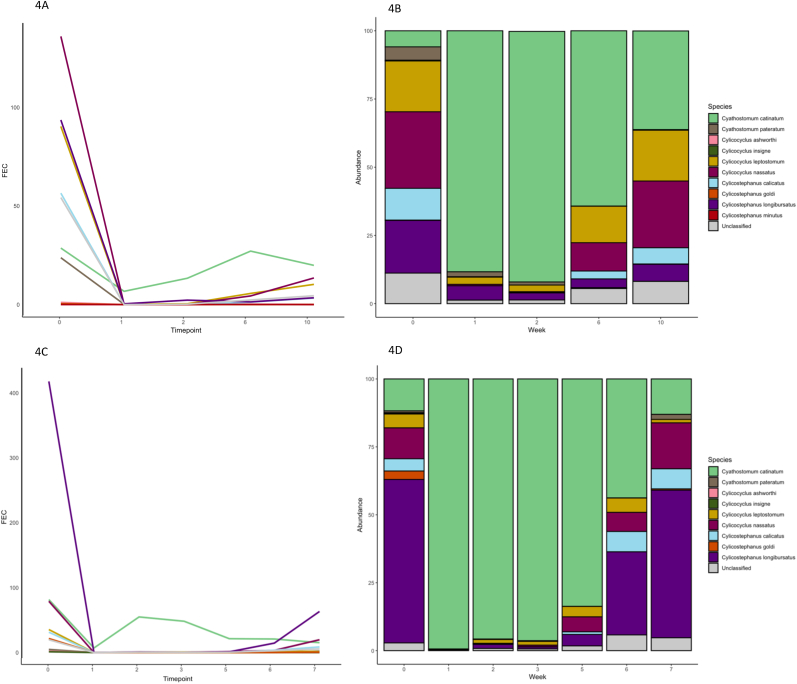
Mares on stud A treated with ivermectin and moxidectin. Resistance was not detected in the mares. The week 1, 2,3 post treatment species are only from 2/3 individuals. The line graph shows the estimated change in FEC for each species and the stacked bar plot shows species abundance. 4A and 4B- stud A mares treated with MOX. 4C and 4D-stud A mares treated with moxidectin.

Mares on stud A did not meet criteria for ML resistance using the criteria from the new WAAVP guidelines for diagnosing anthelmintic resistance using FECRT method (Kaplan et al., 2023), but there were two mares with positive FEC after treatment, and *Cya. catinatum* was the most abundant species in these samples (Fig. 4).

## 4 Discussion

This was the first study to use ITS2 amplicon sequencing to define the relative abundance cyathostomin species composition pre and post treatment in a UK anthelmintic resistant cyathostomin population. There were 12 cyathostomin species identified from the yearlings on the four TB studs sampled, specifically, nine on stud A, eight on stud B, 12 on stud C and eight on stud D (Table 1). These numbers are comparatively lower than the number of species documented from nemabiome studies in wild equine populations or those rarely administered anthelmintics (Poissant et al., 2021), which may indicate that anthelmintic use reduces the diversity of cyathostomin populations by selecting for more resilient and/or prevalent species. 10.13039/100028090Wider evidence to support this hypothesis is difficult to obtain given that, often, there are no data to determine the frequency of treatments in study populations. For example, studies in managed horses in Wales, Thailand and Sweden all had higher numbers of cyathostomin species than this current study, but there were no details of relative treatment frequency for comparison (Mitchell et al., 2019; Halvarsson et al., 2024; Hamad et al., 2024). The most common cyathostomin species found on the four studs in this study were *Cya. catinatum*, *Cyl. nassatus*, *Cyl. longibursatus* and *Cylicocyclus leptostomum.* This result is consistent with data showing that *Cya. catinatum*, *Cyl. nassatus* and *Cyl. longibursatus* together comprised ∼66% of the total cyathostomin populations across all datasets in a review of 37 cyathostomin species reports between 1975 and 2020 (Bellaw and Nielsen, 2020).

In this current study some of the most noteworthy findings were on stud A, where the nemabiome of an anthelmintic resistant population was defined before and after treatment. Following treatment with MLs *Cya. catinatum* was selected for and, in some cases, did not appear to reduce at all two weeks post-treatment; whilst on the same stud, PYR appeared to fail against *Cyl. nassatus* (Fig. 2). Individual species FECR, based on metabarcoding data, revealed *Cya. catinatum* as resistant to MLs, with a FEC reduction of between 34.9 and 59.1% in treatment groups, and *Cyl.* nassatus as resistant to PYR with a 66.7% FEC reduction (Table 2). This suggests that anthelmintic resistance on stud A was a species-specific phenomenon in cyathostomins and raises questions as to whether some species are more likely to develop resistance than others or, alternatively, whether the association between species and resistance is purely stochastic. To our knowledge, the nemabiome tool has only been applied to one other AR study of MLs in Australia (Abbas et al., 2024). This study analysed species abundance after administering various anthelmintic drugs on 22 farms and found AR to MLs on several farms. Nemabiome sequencing revealed that multiple species were responsible for ML resistance across all farms, though individual farm data was not provided. This suggests that anthelmintic resistance may occur stochastically in any species. However, without individual farm data, it is unclear whether individual farms had species-specific AR profiles. Our study would suggest that it is possible that individual farms have species-specific anthelmintic resistant populations. Evidently, further work is needed to define the relationship between anthelmintic resistance and cyathostomin species.

When considering our findings from a clinical perspective, we hypothesise that the use of MLs and PYR in combination on stud A may have resulted in full reduction in egg counts post treatment. Indeed, species specific anthelmintic resistance may be the reason why combination treatments have shown full efficacy against multidrug resistant populations in previous studies (Nielsen et al., 2020); and highlights the potential of combination anthelmintic use, guided by accurate species information, as a strategic approach to treat anthelmintic resistant cyathostomins. Data from simulated models has also predicted that combination anthelmintics may increase the longevity of anthelmintic drugs prior to the development of multidrug resistance (Scare et al., 2020). On the other hand, a previous study found that repeated combination worming was unsustainable, as observed efficacy dropped with each combination treatment over time (Scare et al., 2018). We hypothesise that combination worming will make things worse if the same species are resistant to different anthelmintics, which is possibly what occurred in Scare et al. (2018). In summary, combination worming against cyathostomins should be monitored carefully, and ideally be used together with species level diagnostics to ensure that different species are resistant to different drugs.

A further finding of significance in this study, was the apparent transfer of anthelmintic resistant *Cya. catinatum* between cohorts which was seen on stud A between the March 2021 sampling and January 2022. The two cohorts of weanlings were not co-grazed at any time, however, weanlings sampled in 2022 moved to the pasture previously grazed by the yearlings of 2021 and could have been infected by L3 from the anthelmintic resistant cyathostomins excreted on the pasture during the previous grazing season (Fig. 1). These data highlight the essential need to use rotational grazing alongside other pasture management strategies such as grazing ruminants, resting pastures, and faeces removal. Notably, avoiding the repetitive use of the same pasture for youngstock from year to year appears to be a crucial measure to mitigate the spread of anthelmintic resistance.

The mares on stud A did not meet criteria for ML resistance using the new WAAVP guidelines for diagnosing anthelmintic resistance using FECRT method (Kaplan et al., 2023), but it was noted that there were a small number of mares with positive FECs after treatment, and in these samples *Cya. catinatum* was also the most abundant species post treatment. This suggests these mares were also infected with anthelmintic resistant *Cya. catinatum.* The mares were grazed separately from the yearlings, but pastures were sometimes used for both groups suggesting that intermittent grazing of pasture contaminated with anthelmintic resistant cyathostomins can lead to their spread - which also supports the fact that frequent movement of horses between studs will likely be spreading these parasites.

We did not find anthelmintic resistance on the cyathostomin populations sampled on studs B, C, and D, however, all had reduced ERP's of between 4 and 7 weeks. Nemabiome analysis displayed a mix of species both pre and post treatment with MLs, with the most abundant species pre-treatment tending to recur in greater abundance post-treatment. Similar observations have been made in previous studies using semi-quantitative tools such as the RLB (Molena et al., 2018), supporting a theory that reduced ERPs are not associated with specific species in the same way that we have shown anthelmintic resistance to be on stud A. However, in contrast, another study found species bias post treatment in a case of shortened ERP, where *Cyl. nassatus* was found to be over 50% of all specimens recovered at five weeks post MOX treatment (Nielsen et al., 2022a).

This inconsistency between studies may support the theory that reduced ERPs are caused by a separate, more general, mechanism when compared with anthelmintic resistance (Neilsen et al., 2023), e.g., upregulation of drug efflux exporters which has been shown to occur in anthelmintic resistant cyathostomins (Peachey et al., 2017) (Fig. 3). Further work is needed to draw meaningful conclusions in this area, particularly with respect to early ERPs.

This study is the first investigation into the species responsible for treatment failures of a ML and PYR resistant population in a UK. Our findings suggest that *Cya. catinatum* could explain the ML treatment failures in this population, while *Cyl. nassatus* was implicated in PYR failure. Understanding the mechanisms driving resistance development in these species necessitates further investigation to determine why certain species develop resistance while others remain susceptible to the same drug in the same populations. These studies will help ascertain whether *Cyl. catinatum* is more widely responsible for ML resistance in other cyathostomin populations, whether prevalent species are more prone to developing anthelmintic resistance or whether the emergence of resistance is entirely stochastic, yielding variable results across cases. From a clinical perspective, using drug specific faecal egg count reduction tests and nemabiome analysis together would be a sensible management option going forward to determine which species are susceptible to which anthelmintics. This may allow targeting of specific species in situations where full efficacy is desirable, e.g., during quarantine or in cases of clinical disease; although caution should be exercised before using this as a herd level tool until further research has been conducted to support the sustainability of this approach.

## 5 Conclusions

This study looked at cyathostomin species pre and post treatment in a population with PYR, IVM and MOX resistance. The identification of distinct species associated with resistance to specific anthelmintics raises further questions, particularly regarding results in other anthelmintic resistant populations. These data suggest the potential transmission of AR cyathostomin species between cohorts highlights the need for rigorous management practices to reduce the transfer of anthelmintic-resistant parasites, particularly in the TB breeding industry where animal movement is widespread.

## CRediT authorship contribution statement

**K.E. Bull:** Writing – original draft, Methodology, Investigation, Formal analysis, Data curation. **J. Hodgkinson:** Writing – review & editing, Supervision, Methodology, Conceptualization. **K. Allen:** Writing – review & editing, Supervision, Project administration. **J. Poissant:** Writing – review & editing, Supervision, Methodology. **L.E. Peachey:** Writing – review & editing, Supervision, Methodology, Funding acquisition, Conceptualization.

## Funding

Funding for this project was provided by the 10.13039/501100001280Horserace Betting Levy Board.

**Table 1:** The number of raw pairs of reads generated by the Illumina MiSeq and the number left at each stage of the metabarcoding bioinformatics pipeline for pools of between 2000 and 4000 L3s isolated from faeces of mares and yearlings from the four studs (A-D).

Stud	Age	Timepoint	Drug	Sample ID	Input	filtered	denoisedF	denoisedR	merged	nonchim	*n* Species ≥80%
**A**	**Y**	**0**	**I**	**1**	13545	13219	13207	13134	12580	11554	10
**B**	**Y**	**0**	**M**	**10**	14955	14552	14535	14481	13699	12471	11
**C**	**Y**	**0**	**I**	**11**	12217	11947	11937	11878	11412	10024	10
**C**	**Y**	**4**	**I**	**12**	10719	10425	10420	10379	10290	9793	9
**A**	**Y**	**0**	**P**	**23**	21193	20592	20558	20458	19754	17596	8
**A**	**M**	**0**	**I**	**28**	22647	21982	21942	21839	20827	18684	10
**A**	**M**	**1**	**I**	**29**	19905	19425	19420	19281	19077	18938	4
**A**	**Y**	**3**	**I**	**3**	14504	14173	14172	14082	13881	13735	8
**A**	**M**	**2**	**I**	**30**	40829	39778	39760	39481	38986	38459	7
**A**	**M**	**3**	**I**	**31**	42933	41916	41895	41617	41084	40145	7
**A**	**M**	**5**	**I**	**32**	30809	30014	29992	29818	29390	28115	6
**A**	**M**	**6**	**I**	**33**	14005	13645	13615	13567	13089	11393	9
**A**	**M**	**7**	**I**	**34**	19866	19302	19252	19204	18341	15637	8
**A**	**Y**	**2**	**P**	**35**	16849	16406	16388	16350	16139	15500	10
**A**	**M**	**0**	**M**	**36**	14075	13635	13614	13572	11859	11091	10
**A**	**M**	**1**	**M**	**37**	18849	18450	18447	18337	18127	17377	8
**A**	**M**	**2**	**M**	**38**	16037	15641	15633	15544	15374	15193	8
**A**	**M**	**6**	**M**	**39**	43394	42359	42285	42102	41131	34995	8
**A**	**Y**	**0**	**M**	**4**	16467	16059	16015	15924	15400	13228	8
**A**	**M**	**10**	**M**	**40**	7759	7552	7545	7504	7258	6848	10
**A**	**Y**	**1**	**M**	**5**	29023	28225	28205	27976	27965	27469	3
**A**	**Y**	**2**	**M**	**6**	25232	24598	24595	24456	24440	24111	4
**A**	**Y**	**3**	**M**	**7**	15415	15062	15057	14952	14717	14083	7
**A**	**Y**	**4**	**M**	**8**	11192	10921	10911	10830	10675	10517	6
**B**	**Y**	**6**	**M**	**9**	13363	13026	13019	12936	12716	12271	7
**A**	**Y**	**0**	**I**	**47**	16943	16568	16515	16433	15810	14028	7
**D**	**Y**	**6**	**M**	**56**	21721	21150	21131	21013	19886	18458	11
**A**	**Y**	**2**	**I**	**48**	56271	54459	54348	54057	53583	50687	8
**C**	**Y**	**0**	**M**	**49**	62398	60407	60322	60008	59594	49925	13
**C**	**Y**	**4**	**M**	**50**	45008	43536	43471	43209	42817	36704	10
**C**	**Y**	**6**	**M**	**51**	48507	47016	46968	46727	46500	43851	11
**B**	**Y**	**0**	**I**	**52**	51681	50011	49974	49682	49468	45500	11
**B**	**Y**	**7**	**I**	**53**	47230	45594	45564	45232	45026	39057	8
**D**	**Y**	**0**	**M**	**54**	48480	46951	46905	46604	46345	42501	9
**D**	**Y**	**4**	**M**	**55**	79595	76970	76862	76547	76086	66421	8
**D**	**Y**	**0**	**I**	**75**	62507	59412	59331	58786	57899	57899	9
**D**	**Y**	**4**	**I**	**76**	51233	48294	48294	47655	47309	47309	10

## Declaration of competing interest

None.

## Abbreviations

FBZ: Fenbendazole
IVM: Ivermectin
MOX: Moxidectin
PYR: Pyrantel
ML: Macrocyclic lactone
FECRT: Faecal egg count reduction test
ERP: Egg reappearance period
TB: Thoroughbred
